# Effect of levosimendan in experimental verapamil- induced myocardial depression

**DOI:** 10.1186/1757-7241-18-12

**Published:** 2010-03-11

**Authors:** Jouni Kurola, Heli Leppikangas, Jarkko Magga, Leena Lindgren, Vesa Kiviniemi, Juha Rutanen, Esko Ruokonen

**Affiliations:** 1 Department of Intensive Care, Kuopio University Hospital, Kuopio, Finland; 2 Department of Anesthesiology, Tampere University Hospital, Tampere, Finland; 3 IT Centre, Kuopio University, Kuopio, Finland

## Abstract

**Background:**

Calcium antagonist overdose can cause severe deterioration of hemodynamics unresponsible to treatment with beta adrenergic inotropes. The aim of the study was to evaluate in an experimental model the effects of levosimendan during severe calcium antagonist intoxication.

**Methods:**

Twelve landrace-pigs were intoxicated with intravenous verapamil at escalating infusion rates. The infusion containing 2.5 mg/ml verapamil was used aiming to a reduction of cardiac output by 40% from the baseline value. Intoxicated pigs were randomized into two groups: control (saline) and levosimendan (intravenous bolus). Inotropic effect was measured as a change in a maximum of the positive slope of the left ventricular pressure (LV dP/dt). The survival and hemodynamics of the animals were followed for 120 min after the targeted reduction of cardiac output.

**Results:**

In the control group, five out of six pigs died during the experiment. In the levosimendan group, one pig died before completion of the experiment (p = 0.04). In the levosimendan group a change in LV dP/dt was positive in four out of six pigs compared to one out of six pigs in the control group (p = ns).

**Conclusions:**

In this experimental model, the use of levosimendan was associated with improved survival.

## Background

In the year 2004 more than 10000 toxic exposures to calcium channels blockers were reported in the United States. Of those exposures, 3.3% were associated with severe bradycardia, hypotension and acute negative inotropy. Altogether, there were 62 (0.6%) deaths due to calcium channel blocker overdoses. Verapamil and diltiazem involved the majority of these fatal poisonings [1]. The majority of the exposures occurred accidentally (79%), but a significant (18%) part was suicide attempts. Moreover, a small amount of overdoses was in children or due to iatrogenic treatments [1]. The number of toxic incidents is increasing [2,3].

Calcium channel blocker overdose causes intractable hypotension, bradycardia, cardiac conduction abnormalities and depression of myocardial contractility, leading to central nervous system (e.g. syncope, seizures and coma), respiratory (non-cardiogenic pulmonary edema) and metabolic (e.g. hyperglycemia and acidosis) disorders [4]. The management of calcium channel blocker poisoning includes the use of a wide variety of medications and also non-pharmacological techniques [4]. The aims are to support vital functions and, on the other hand, to prevent the further absorption of calcium channel blockers from the gut with lavage and activated charcoal. The management of the cardiovascular symptoms is focused on normalization of sinus rate by atropine as well as pacing and restoration of normal arterial pressure (plasma volume expanders and catecholamines). The negative inotropy can be partly reversed by using β-adrenergic agonists, phosphodiesterase inhibitors, glucagon, insulin with dextrose and calcium salts [5]. Also a case report regarding the use of levosimendan has been published [6].

Both verapamil and diltiazem decrease myocardial contractility [7] at high plasma concentrations, as seen in acute poisoning [8]. The negative inotropy caused by these drugs is due to a direct cardiac effect, shown in vitro in Langendorff perfused isolated hearts [9]. The sustained effect of verapamil may be related to its active hepatic metabolite, nor-verapamil, which has 50% of the potency of the parent compound [10]. The symptoms of calcium channel blocker overdose do not always respond to treatment with conventional beta adrenergic drugs. A rather new calcium sensitizer, levosimendan, is targeted to treatment of acute decompensated heart failure. Levosimendan induces a positive inotropic effect mediated through calcium-dependent binding of the compound to troponin C [11,12]. This mechanism of action increases sensitivity of contractile proteins for calcium. Levosimendan works also under extreme conditions e.g. acidosis [13] and sepsis [14]. Levosimendan also causes coronary dilation and systemic vasodilatation [15] through opening of ATP-sensitive potassium channels [16].

The aim of our study was to assess the effects of levosimendan in experimental porcine poisoning model of severe verapamil intoxication.

## Methods

National Animal Ethics Committee of Finland approved the method. The animal care, welfare and procedures were carried out in accordance with the regulations of the Council of Europe.

### Animals and anesthesia

Twelve [12] landrace- pigs (28 ± 5 kg) were deprived of food, but not water 12 h before the experiments. Premedication with medetomidine 50 μg/kg, ketamine 10 mg/kg and fentanyl 5 μg/kg intramuscularly was followed by cannulation of an ear vein and intravenous administration of 2 mg/kg of propofol for tracheotomy. Anesthesia was maintained with propofol (10 mg/kg/hour) and fentanyl (30 μg/kg/hour). The animals were ventilated with a volume-controlled mode (Servo 900, Siemens, Elema AB, Solna Sweden) with 5 cmH_2_O of positive end-expiratory pressure (PEEP). FIO_2 _(0.3-0.6) was adjusted to keep PaO_2 _levels between 13.3 kPa to 20 kPa. Tidal volume was kept at 10 ml/kg, and the minute ventilation was adjusted to maintain PaCO_2 _levels between 4.5 to 5.5 kPa.

### Animal preparation

A fluid-filled catheter was inserted into the right femoral artery (single-lumen central venous catheter, Arrow, Arrow International Inc, Reading, PA) and a pulmonary artery catheter (7.5F flow-directed, Arrow, Arrow International Inc, Reading, PA) introduced via the right internal jugular vein. The angiography (Impulse™, Boston Scientific, USA) catheter was inserted into left ventricle via left femoral artery to measure a change in a maximum of the positive slope of the left ventricular pressure (LVdP/dt). During instrumentation, the animals received 5 ml/kg/h infusions of 0.9% saline and gelatin (Gelofusine^®^, B. Braun Medical, Germany). Additional fluid was administered if necessary to keep pulmonary artery occlusion pressure (PAOP) between 5 and 8 mmHg. Body temperature of the animals was kept above 38°C using an operating table heater and warmed fluids.

### Experimental protocol

After instrumentation, a stabilization period of at least 30 minutes was allowed followed by the baseline measurements. Verapamil intoxication was then induced by a long-lasting intravenous infusion containing 2.5 mg/ml of verapamil at an escalating rate into the right internal jugular vein. The rate of verapamil infusion was increased by 2.5 ml/h in every 15 minutes. The infusion was targeted to decrease cardiac output 40% from the baseline value. The administration and the amount of verapamil were based on a pilot trial in three pigs.

At completion of the verapamil intoxication phase, both the control and the levosimendan groups received a continuous infusion of verapamil 12.5 mg/h to maintain the toxicity level. Thereafter, animals in the control group received 25 ml bolus of saline in 15 minutes and the levosimendan group 1.25 mg levosimendan (Simdax^®^, Orion Pharma, Espoo, Finland) in the same volume and time. Arterial blood samples were obtained in heparinezed tubes at Intox 0 and in the end of experiment for measurement of plasma concentrations of verapamil and nor-verapamil, calcium, lactate, sodium, potassium and glucose. The analytical method used was liquid chromatography-mass spectrometry. At the end of experiment, the surviving animals were killed with a high dose of verapamil. The total dose of verapamil given to the pigs was recorded at the end of the experiment.

### Hemodynamic monitoring

Left ventricular pressure (LVP), mean arterial pressure (MAP), central venous pressure (CVP), end diastolic pressure (EDP) and pulmonary artery occlusion pressure (PAOP) were recorded with quartz pressure transducers and displayed continuously on a multimodular monitor (S/5 Compact Critical Care Monitor, Datex-Ohmeda™, Helsinki, Finland). All pressure transducers were calibrated simultaneously and zeroed to the level of the heart. The inotropic effect was measured as a change in a maximum of the positive slope of the left ventricular pressure (LV dP/dt). LV dP/dt was measured once a minute which represent a mean value over one minute cardiac cycles. A mean value of 5 minutes was recorded and its coefficient of variation in the control group was 3.6% (2.2; 5.8) and 4.1% (1.8; 10.5) in the levosimendan group (ns). Cardiac output (CO) was measured by a thermodilution technique and mean value of three measurements was used with room temperature saline injectates of 5 ml. (Datex-Ohmeda™, Helsinki, Finland). Heart rate (HR) was measured from the continuously monitored ECG.

### Statistical analysis

Mann-Whitney test was used to analyze differences in hemodynamic and laboratory measures at preintoxication (baseline) and postintoxication (from Intox 0 to Intox 120) phases. Values are presented as median and interquartale range. Statistical analyses were done using a statistical program SPSS for Windows version 14.0 (SPSS^® ^Inc. Chicago, USA). P-values of less than 0.05 were considered statistically significant.

## Results

Baseline data on hemodynamics and the laboratory values of calcium and lactate are presented in Tables 1 and 2. There was no difference between the groups in baseline data excluding hemoglobin, which was higher in the levosimendan group 73 (68; 73) g/l vs. 65 (66; 73) g/l (p = 0.04).

**Table 1:** Comparison of hemodynamic values (median, IQR) between groups before verapamil infusion (Baseline) and at the time when intoxication was complete (Intox 0) (p = ns between levosimendan and control groups in both baseline and intox 0).

	Baseline	Intox 0
**MAP**		

control	95 (75;103)	43 (39;50)

levosimendan	100 (93;106)	44 (44;55)

**CO**		

control	4.6 (3.4;5.5)	2.4 (1.9;2.8)

levosimendan	4.4 (4.1;4.7)	2.1 (2.0;2.2)

**LV dP/dt (mmHg/s)**		

control	1730 (1548;1901)	761 (609;778)

levosimendan	2096 (2014;2238)	624 (518;736)

**HR (beats/min)**		

control	111 (98;132)	84 (78;94)

levosimendan	103 (95;111)	85 (72;93)

**CVP (mmHg)**		

control	6 (5;6)	7 (7;8)

levosimendan	5 (4;7)	7 (5;8)

**EDP (mmHg**)		

control	19 (12;23)	13 (11;15)

levosimendan	15 (13;16)	15 (12;16)

**Table 2:** Comparison of calcium and lactate values (median, IQR) between groups before verapamil infusion (Baseline), at the time when intoxication was complete (Intox 0) and right before clinically estimated collapse of hemodynamics (End of experiment) (p > 0.05 between the groups).

	Baseline	Intox 0	End of experiment
**Ca (mmol/l)**			

control	1.20 (0.98;1.30)	1.10 (1.01;1.38)	1.06 (0.85;1.21)

levosimendan	1.02 (0.96;1.27)	1.00 (0.89;1.11)	0.98 (0.84;1.15)

**Lactate (mmol/l)**			

control	0.8 (0.6;0.7)	1.9 (1.1;1.9)	6.6 (4.7;8.7)

levosimendan	0.6 (0.6;0.7)	1.1 (0.9;1.4)	6.7 (1.2;8.6)

In each animal, cardiac output decreased by 40% as planned. In the planned control group the reduction was 45% (43; 54) and in the planned levosimendan group 49% (44; 50) (p = ns). The dose of verapamil required to induce toxicity was 22 (22; 37) mg and it took 53 (45; 71) minutes in the future control group and 22 (16; 29) mg and 53 (34; 60) minutes in the future levosimendan group (p = ns). Total amount of verapamil infused for both intoxication and maintainance period was 42 (38; 46) mg in the control group and 47 (46; 50) mg in levosimendan group (p = ns). Plasma concentrations of verapamil and nor-verapamil at Intox 0 were in the levosimendan group 238.0 (222.0; 385.0) ng/ml and 3.0 (2.5; 8.1) ng/ml compared to the control group 293.5 (217.5; 365.5) ng/ml and 5.1 (2.2; 8.4) ng/ml (p = ns), respectively. There were no differences between levosimendan and control groups in verapamil and nor-verapamil concentrations between groups at the end of experiment 279.5(226.0; 315.0) ng/ml and 11.3 (10.4; 13.7) ng/ml vs. 300.0 (223.0; 339.8) ng/ml and 9.6 (9.8; 14.3) ng/ml (p = ns), respectively.

The hemodynamic and the laboratory data of lactate and calcium at the time point when intoxication was complete (Intox 0) are presented in Tables 1 and 2, and there were no differences between groups. There were no differences between groups in laboratory values at the end of the experiment (p = ns). The laboratory values of sodium, potassium and glucose were comparable between groups throughout the experiment (p = ns).

**Figure 1:**
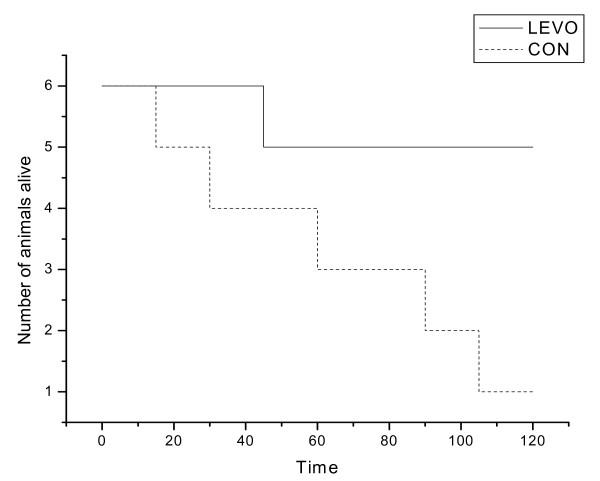
**Kaplan-Meier survival curve in levosimendan (LEVO) and control (CON) groups**.

Five out of six pigs died during the experiment in the control group. In the levosimendan group one pig died before completion of the experiment. The median time alive from the completion of intoxication was 75 (60; 101) minutes in the control group and 120 (120; 120) minutes in the levosimendan group, respectively. The Kaplan- Meier survival curve is presented in Figure 1 (p = 0.04).

**Figure 2:**
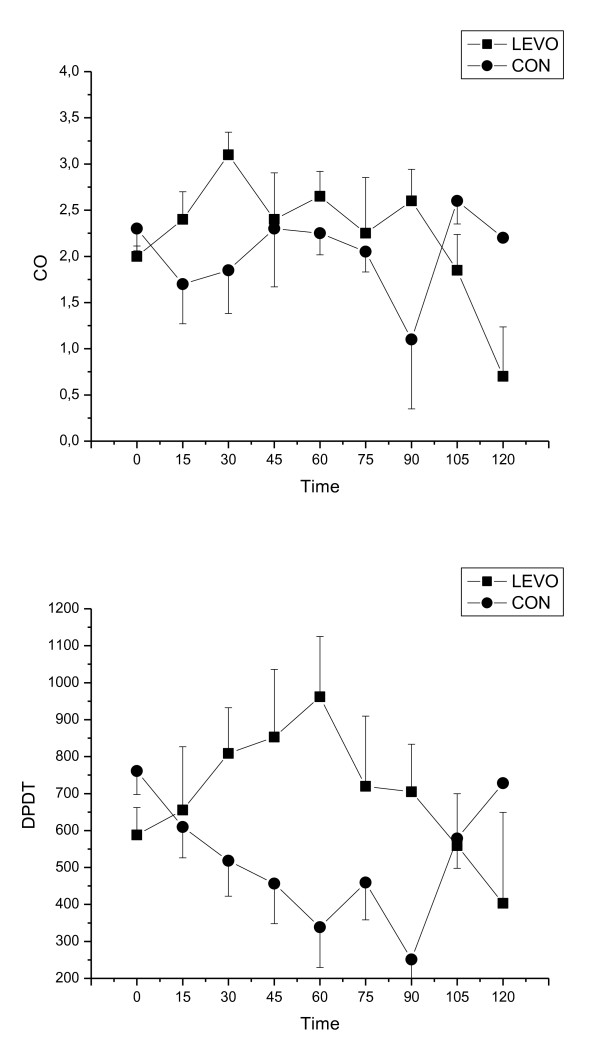
**Cardiac output (CO) and Maximum of the positive slope of the left ventricular pressure (LV dP/dt) for control (CON) and levosimendan (LEVO) groups(median ± IQR) versus time (in minutes)**.

**Figure 3:**
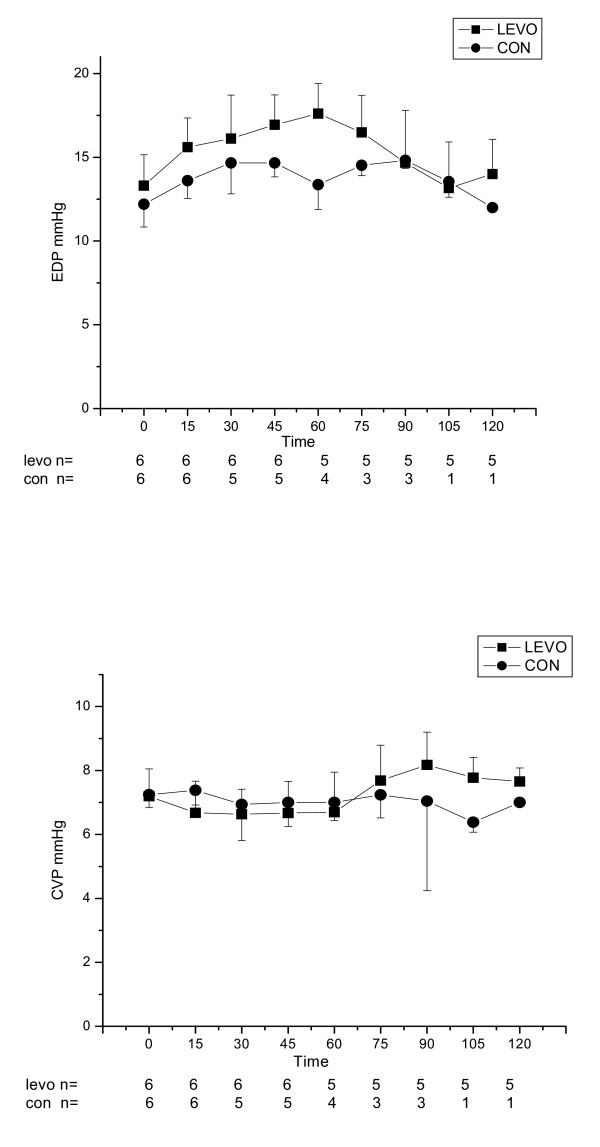
**End diastolic pressure (EDP, mmHg) and central venous pressure (CVP, mmHg), for control (CON) and levosimendan (LEVO) groups (median ± IQR) versus time (in minutes)**. Number of surviving animals is presented under the x- axis at each time point.

**Figure 4:**
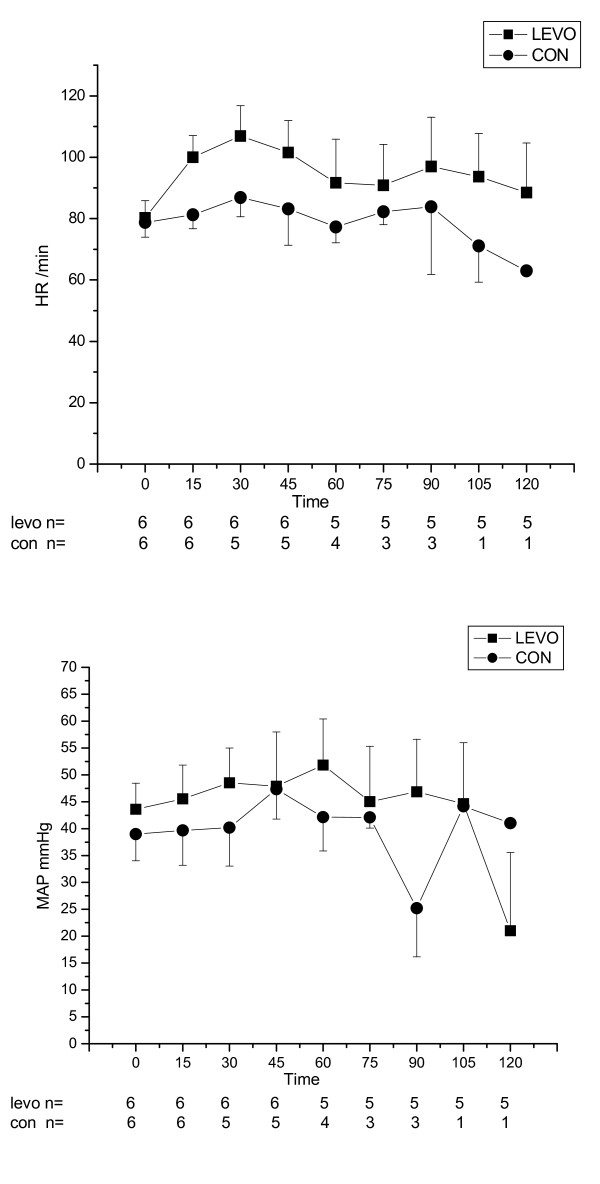
**Heart rate (HR, beats/min) and mean arterial pressure (MAP, mmHg) for control (CON) and levosimendan (LEVO) groups (median ± IQR) versus time (in minutes)**. Number of surviving animals is presented under the x- axis at each time point.

After completion of intoxication, the group receiving levosimendan had a tendency towards higher LV dP/dt than the control group, however there were no statistically significant differences either in LV dP/dt, CO, HR, MAP, CVP and EDP between groups (Figure 2, 3 and 4).

## Discussion

The main finding of our study was that levosimendan improved survival in severe verapamil intoxication. In our experimental model, verapamil resulted in negative inotropy in control group assessed with dPdT. In contrast, heart rate was less prominently affected.

Verapamil intoxication is related to its intended action on myocardial and smooth muscle cells, where it competitively blocks cell surface slow calcium channels. Inhibition of calcium influx is responsible for depression of contractility causing a myocardial stunning-like syndrome [17-20]. The function and mechanical efficiency of stunned myocardium is depressed due to decreased sensitivity of the myofibrils to calcium [18].

Levosimendan enhances cardiac contraction by improving the use of available calcium rather by inundating the cell with excessive calcium [21]. The use of traditional inotropes is associated with increased energy consumption and arrhythmogenesis due to elevated intracellular calcium concentration leading to apoptosis in long term use [22,23]. Levosimendan causes vasodilatation via opening of adenosine triphosphatase-sensitive K^+ ^channels [24]. This effect may contribute to coronary [25] and systemic [26] vasodilatation with the intravenous administration of levosimendan.

The levosimendan bolus was well tolerated. Even though the vasodilating effect of levosimendan has been well documented [26-28] it is noteworthy that it did not have deleterious effect on mean arterial pressure. It is conceivable that the inotropic effect of levosimendan was more prominent than the vasodilating effect.

Inotropic effect was measured as a change in a maximum of the positive slope of the left ventricular pressure (LV dP/dt). In the levosimendan group LV dP/dt increased by 38% from the baseline to the Intox 60 minutes, whereas LV dP/dt decreased in the control group by 31% during the same time interval. The same trend was seen for CO, but due to the small number of surviving animals in the control group, a significant difference was not reached between study groups. There were no clinical or statistical differences in HR, MAP, CVP and EDP between the study groups.

The first limitation of the study is that an animal model is not exactly like the toxicity seen in human beings. We chose a pig model because it has been used in previous studies of verapamil toxicity [29], and pigs have similar cardiovascular systems as humans [30]. The second limitation is the small number of animals and the survival rate was very low in control group; therefore detailed statistical analysis of hemodynamic differences between the groups was not possible. The third limitation is the use of intravenous verapamil as a substitute for oral ingestion that can prolong the absorption of verapamil. This limitation was minimized by continuing the verapamil infusion throughout the study to mimic oral ingestion. On the other hand, concentrations of verapamil and its active metabolite, nor-verapamil, were about similar in the two study groups. Although oral ingestion might have a different pharmacokinetics, according to hemodynamic data, we induced a severe verapamil poisoning.

In summary, treatment with levosimendan improved survival in pigs severly poisoned with verapamil. Levosimendan seemed to maintain cardiac performance especially during the early phase of intoxication without excessive vasodilatation. Confirmation of the effectiveness of levosimendan for pharmacotherapy of verapamil intoxication in humans requires further experiments.

## Competing interests

The authors declare that they have no competing interests.

## Authors' contributions

JK and HL participated to the design of the study, performed the study and prepared the manuscript. ER designed the study and prepared the manuscript. JR performed the study. JM and LL participated to the design of the study and VK made statistical analysis. All authors read and approved the final manuscript.
